# Draft Genome Sequence of a Kale (*Brassica oleracea* L.) Root Endophyte, *Pseudomonas* sp. Strain C9

**DOI:** 10.1128/genomeA.00163-17

**Published:** 2017-04-13

**Authors:** Aurelie Laugraud, Sandra Young, Emily Gerard, Maureen O’Callaghan, Steven Wakelin

**Affiliations:** a AgResearch, Ltd., Lincoln Science Centre, Christchurch, New Zealand; b Scion Research, Christchurch, New Zealand

## Abstract

*Pseudomonas* sp. strain C9 is a plant growth–promoting bacterium isolated from the root tissue of *Brassica oleracea* L. grown in soil from Marlborough, New Zealand. Its draft genome of 6,350,161 bp contains genes associated with plant growth promotion and biological control.

## GENOME ANNOUNCEMENT

*Pseudomonas* spp. are well known as plant growth–promoting (PGP) bacteria. They are common in (endophytic) and on (rhizoplane) plant roots (1), where they enhance plant growth and protect from disease using a range of mechanisms (2). We isolated *Pseudomonas* sp. strain C9 during a study to identify novel bacterial strains that could be used to improve efficiency in nutrient use (3). The strain was isolated from the roots of *Brassica oleracea* L. (kale, cv. Red Russian) in pasture soil from Marlborough, New Zealand. It is phylogenetically grouped within the *P. mandelii* subgroup of the genus *Pseudomonas* (4).

Genomic DNA was extracted using the ISOLATE II Genomic DNA kit (Bioline, USA) and sequenced on a MiSeq platform (Illumina, USA). Libraries were prepared by sonication with Covaris or Bioruptor to produce fragments of 500 bp in size, extended by Klenow DNA polymerase, and ligated into T-vectors. We used two libraries to sequence this genome; the first had an insert size of 450 bp (paired-end), and the second had an insert size of 8,000 bp (mate-pair). Sequencing produced 18 million reads (~100-fold coverage) of 120 bp each. The fragments were assembled in A5-miseq (5) with default settings and SSPACE (6). The final assembly has seven scaffolds, with a maximum length of 5,775,319 bp. The genome was annotated using the NCBI Prokaryotic Genome Annotation Pipeline (7).

The draft genome of *Pseudomonas* sp. C9 comprises 6,350,161 bp and has a G+C content of 58.9%. A total of 5,764 genes are annotated, encoding 5,689 proteins. The genome has 60 tRNAs and one predicted CRISPR cluster. Genes associated with antibiotic production and fungal suppression, such as the PhzF phenazine biosynthesis protein and hydroxyphenazine, were found. We also found PtrA, a LysR-type transcriptional regulator of genes required for antifungal biocontrol activity (8). The GacS/GacA two-component signal transduction system important in fungal antagonism (9) was also present in the genome, which is in common with other biocontrol and plant growth–promoting strains of *Pseudomonas* spp. The genome also possesses four siderophores genes and enzymes (siderophore-iron reductase, Fe-S cluster protein, siderophore biosynthesis protein SbnG, NADPH-dependent ferric siderophore reductase, and iron-siderophore ABC transporter permease), enabling the bacterium to scavenge and compete for iron (10).

### Accession number(s).

Contigs from the assembled genome of *Pseudomonas* sp. C9 were deposited at GenBank under the accession number MPAK00000000. This version described here is the first version, MPAK01000000.

## References

[1] Rosenblueth M, Martínez-Romero E 2006 Bacterial endophytes and their interactions with hosts. Mol Plant Microbe Interact 19:827–837. doi:10.1094/MPMI-19-0827. 16903349

[2] Raaijmakers JM, Paulitz TC, Steinberg C, Alabouvette C, Moënne-Loccoz Y 2009 The rhizosphere: a playground and battlefield for soilborne pathogens and beneficial microorganisms. Plant Soil 321:341–361. doi:10.1007/s11104-008-9568-6.

[3] Wakelin SA, Young S, Gerard E, Mander C, O’Callaghan M 2017 Isolation of root-associated *Pseudomonas* and *Burkholderia* spp. with biocontrol and plant growth promoting traits. Biocontrol science and technology. Biocontrol Sci Technol 27:139–143. doi:10.1080/09583157.2016.1248899.

[4] Gomila M, Peña A, Mulet M, Lalucat J, García-Valdés E 2015 Phylogenomics and systematics in *Pseudomonas*. Front Microbiol 6:214. doi:10.3389/fmicb.2015.00214. 26074881 PMC4447124

[5] Coil D, Jospin G, Darling AE 2014 A5-miseq: an updated pipeline to assemble microbial genomes from Illumina MiSeq data. Bioinformatics 31:587–589. doi:10.1093/bioinformatics/btu661. 25338718

[6] Boetzer M, Henkel CV, Jansen HJ, Butler D, Pirovano W 2011 Scaffolding pre-assembled contigs using SSPACE. Bioinformatics 27:578–579. doi:10.1093/bioinformatics/btq683. 21149342

[7] Angiuoli SV, Gussman A, Klimke W, Cochrane G, Field D, Garrity G, Kodira CD, Kyrpides N, Madupu R, Markowitz V, Tatusova T, Thomson N, White O 2008 Toward an online repository of standard operating procedures (SOPs) for (meta)genomic annotation. Omics 12:137–141. doi:10.1089/omi.2008.0017. 18416670 PMC3196215

[8] Shah N, Klaponski N, Selin C, Rudney R, Dilantha Fernando WG, Belmonte MF, de Kievet TR 2016 PtrA is functionally intertwined with GacS in regulating the biocontrol activity of *Pseudomonas chlororaphis* PA23. Front Microbiol 7:1512. doi:10.3389/fmicb.2016.01512. 27713742 PMC5031690

[9] Heeb S, Haas D 2001 Regulatory roles of the GacS/GacA two component system in plant-associated and other Gram-negative bacteria. Mol Plant Microbe Interact 14:1351–1363. doi:10.1094/MPMI.2001.14.12.1351. 11768529

[10] Kloepper JW, Schroth MN 1981 Relationship of in vitro antibiosis of plant growth-promoting rhizobacteria to plant growth and the displacement of root microflora. Phytopathology 71:1020–1024. doi:10.1094/Phyto-71-1020.

